# Integrative machine learning and bioinformatics analysis to identify cellular senescence-related genes and potential therapeutic targets in ulcerative colitis and colorectal cancer

**DOI:** 10.3389/fbinf.2025.1599098

**Published:** 2025-07-28

**Authors:** Tianle Xue, Yunpeng Chen, Xiaomeng Li, Zhixiang Zhou, Qiyang Chen

**Affiliations:** 1 Strathclyde Institute of Pharmacy and Biomedical Sciences, University of Strathclyde, Glasgow, United Kingdom; 2 China Pharmaceutical University, Nanjing, China

**Keywords:** cellular senescence, ulcerative colitis, colorectal cancer, integrative machine learning, immune infiltration, therapeutic targets

## Abstract

**Background:**

Ulcerative colitis (UC) is a chronic inflammatory condition that predisposes patients to colorectal cancer (CRC) through mechanisms that remain largely undefined. Given the pivotal role of cellular senescence in both chronic inflammation and tumorigenesis, we integrated machine learning and bioinformatics approaches to identify senescence‐related biomarkers and potential therapeutic targets involved in the progression from UC to CRC.

**Methods:**

Gene expression profiles from six GEO datasets were analyzed to identify differentially expressed genes (DEGs) using the limma package in R. Weighted gene co-expression network analysis (WGCNA) was employed to delineate modules significantly associated with UC and CRC, and the intersection of DEGs, key module genes, and senescence‐related genes from the CellAge database yielded 112 candidate genes. An integrated machine learning (IML) model—utilizing 12 algorithms with 10-fold cross-validation—was constructed to pinpoint key diagnostic biomarkers. The diagnostic performance of the candidate genes was evaluated using receiver operating characteristic (ROC) analyses in both training and validation cohorts. In addition, immune cell infiltration, protein–protein interaction (PPI) networks, and drug enrichment analyses—including molecular docking—were performed to further elucidate the biological functions and therapeutic potentials of the identified genes.

**Results:**

Our analysis revealed significant transcriptomic alterations in UC and CRC tissues, with the turquoise module demonstrating the strongest association with disease traits. The IML approach identified five pivotal genes (ABCB1, CXCL1, TACC3, TGFβI, and VDR) that individually exhibited AUC values > 0.7, while their combined diagnostic model achieved an AUC of 0.989. Immune infiltration analyses uncovered distinct immune profiles correlating with these biomarkers, and the PPI network confirmed robust interactions among them. Furthermore, drug enrichment and molecular docking studies identified several promising therapeutic candidates targeting these senescence‐related genes.

**Conclusion:**

This study provides novel insights into the molecular interplay between cellular senescence and the UC-to-CRC transition. The identified biomarkers not only offer strong diagnostic potential but also represent promising targets for therapeutic intervention, paving the way for improved clinical management of UC-associated CRC.

## 1 Introduction

Colorectal cancer (CRC) stands as one of the foremost causes of cancer-related morbidity and mortality globally, posing a significant challenge to public health (Arnold et al., 2017; GBD, 2019 Colorectal Cancer Collaborators et al., 2022; Musa and Ali, 2020). Ulcerative colitis (UC), a chronic inflammatory bowel disease, not only drastically impairs patients’ quality of life but also escalates the risk of developing CRC over time (Shah and Itzkowitz, 2022; Yashiro, 2014). Studies have shown that prolonged duration of UC increases the likelihood of CRC occurrence (Dan et al., 2023), with cell senescence playing a pivotal role in the carcinogenic process (Risques et al., 2011). However, at the level of cellular senescence, current research on the key genes of colorectal cancer and ulcerative colitis is not clear, and there is currently no study analyzing the relationship between the two diseases and cell senescence from a genomic perspective. Therefore, the research on related genes and the development of drugs are crucial.

Cell Senescence (CS) is a complex biological process in which the gradual decline in physiological functions increases susceptibility to diseases such as cancer (Kirkland and Tchkonia, 2017; López-Otín et al., 2023). Genes that induce cellular aging often become overexpressed in human tissues with age, and are significantly overexpressed in anti longevity and tumor suppressor genes, while genes that inhibit cellular aging overlap with longevity promoting genes and oncogenes (Aramillo Irizar et al., 2018; Schmitt et al., 2022). Aging cells release pro-inflammatory cytokines and other factors known as senescence associated secretory phenotype (SASP), which lead to chronic inflammation, impaired tissue regeneration, aging, and age-related diseases, like cancer (Ou et al., 2021). Understanding the determinants of cellular aging and its correlation with aging is crucial for dissecting the potential mechanisms of aging and age-related diseases, as well as exploring potential therapeutic pathways.

CellAge is a manually curated database that contains 1279 human genes that drive cellular aging (Avelar et al., 2020). It was compiled after conducting scientific literature searches on gene manipulation experiments in primary, immortalized, or cancer human cell lines that induce or inhibit CS in cells (Chatsirisupac et al., 2019). CellAge aging inducers and inhibitors overlap with oncogenes in the tumor suppressor gene (TSG) database (TSGene 2.0) and ONGene database, and can therefore be used to study cancer-related genes (Zhao et al., 2016; Liu et al., 2017). By excavating deeply into the databases related to cellular senescence, we can gain a more profound understanding of the relevant processes involved in aging and their roles in diseases.

Machine learning (ML) helps humans learn patterns from complex data to predict future behavioral outcomes and trends (Haug and Drazen, 2023). ML is widely used for variable filtering and variable selection (Cascianelli et al., 2023). Previously, research commonly used a single ML algorithm or two integrated ML algorithms (such as artificial neural networks (Eetemadi and Tagkopoulos, 2019), support vector machines (Huang et al., 2018) and gradient boosting machines (Du et al., 2022)) to optimize variables. However, a single or only two integrated ML algorithms may miss important potential genes, while integrated ML (IML) methods have more advantages in variable screening and model construction (Zhang L. et al., 2023). In this study, we focus on studying UC and CRC, using bioinformatics methods combined with IML to investigate in detail the related genes of UC and CRC at the cellular aging level, explore the genetic and transcription factors of UC and CRC, and predict potential therapeutic drugs.

## 2 Methods

### 2.1 Selection of datasets

**FIGURE 1:**
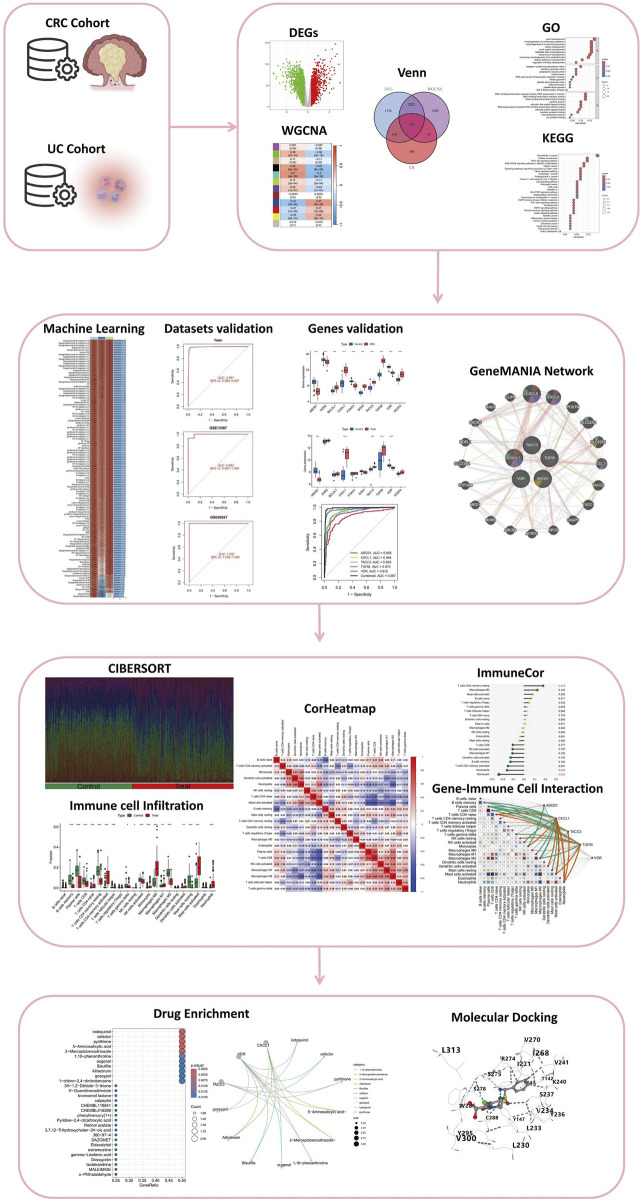
Comprehensive Analysis Workflow for the Study of Colitis-Associated Colorectal Cancer (CRC) Transformation. The workflow includes the analysis of CRC and UC cohorts, identification of differentially expressed genes (DEGs), weighted gene co-expression network analysis (WGCNA), integration through Venn diagrams, and functional enrichment analysis via Gene Ontology (GO) and Kyoto Encyclopedia of Genes and Genomes (KEGG). The study further incorporates machine learning for dataset validation, gene validation using GeneMANIA network analysis, immune cell infiltration assessment through CIBERSORT, and visualization with correlation heatmaps (CorHeatmap). Gene-immune cell interaction is examined using ImmuneCor, followed by drug enrichment analysis and molecular docking to explore therapeutic potentials.

Datasets were downloaded from the NCBI Gene Expression Omnibus (GEO; https://www.ncbi.nlm.nih.gov/geo/) using the keywords “Colorectal Cancer” or “Ulcerative Colitis.” Our data analysis process is demonstrated in Figure 1. Detailed information for each dataset, including microarray platform, sample groups, accession numbers, and sample sizes—was recorded. Only datasets containing colon tissue samples from patients with colorectal cancer and ulcerative colitis were included. A total of 6 datasets, namely, GSE52060 (Medico et al., 2013), GSE87211 (Hu et al., 2018), GSE90627 (Guo et al., 2017), GSE36807 (Montero-Meléndez et al., 2013), GSE53306 (Zhao et al., 2015), and GSE13367 (Bjerrum et al., 2010), were integrated for this study. The training set were selected as GSE52060, GSE87211, GSE36807, and GSE53306. The testing set were selected as GSE90627 and GSE13367. The details for all datasets are presented in Supplementary Table S1. To correct for batch effects from different studies, we used the “ComBat” function in the “sva” package (version 3.5.0) (Johnson et al., 2007; Leek et al., 2012). The effectiveness of batch correction was evaluated by comparing data quality before and after adjustment using principal component analysis (PCA) (Jolliffe and Cadima, 2016).

### 2.2 Identification of differentially expressed genes (DEGs) in UC and CRC

To identify key genetic alterations associated with UC and CRC, we performed differential gene expression analysis between case and control groups using the Linear Models for Microarray Data (limma) package in R. Limma is a widely used statistical tool that applies linear models to gene expression data while leveraging empirical Bayes methods to moderate the standard errors of estimated log-fold changes. This approach enhances the stability of statistical inference, particularly in studies with small sample sizes (Ritchie et al., 2015). To determine significantly differentially expressed genes (DEGs), we utilized the eBayes function, which computes moderated t-statistics, F-statistics, and log-odds of differential expression for each gene. Genes were considered significantly differentially expressed if they met the threshold of a false discovery rate (FDR) < 0.05 (adjusted p-value <0.05) and demonstrated an absolute fold change (FC) greater than 0.585 (|log_2_FC| > 0.5). These stringent criteria helped ensure the robustness and reliability of our findings, highlighting genes with substantial expression changes that may play critical roles in UC and CRC pathogenesis.

### 2.3 Construction of gene Co-expression networks using weighted gene Co-expression network analysis (WGCNA)

To explore functional gene relationships and identify disease-associated modules, we performed Weighted Gene Co-expression Network Analysis (WGCNA). This method constructs gene co-expression networks and detects modules of highly correlated genes, often linked to specific biological traits (Langfelder and Horvath, 2008). As a crucial preprocessing step to ensure a scale-free network topology, we determined the optimal soft-thresholding power (β), selecting a β value where the scale-free topology fit index (R^2^) exceeded 0.8. A minimum module size of 60 genes was set to identify meaningful gene clusters.

Next, the adjacency matrix was transformed into a Topological Overlap Matrix (TOM), which enhances network robustness by reducing the effects of noise and spurious correlations. To identify gene clusters, we calculated the TOM-based dissimilarity measure (1 - TOM) and applied hierarchical clustering to group genes with similar expression patterns into modules. To refine module detection, dynamic tree cutting was implemented to segment the clustering dendrogram. To assess biological relevance, we correlated module eigengenes (principal components of modules) with clinical traits of UC and CRC. Modules with the strongest correlations and lowest p-values were selected for further analysis, helping identify key gene clusters involved in disease mechanisms and potential therapeutic targets.

### 2.4 Acquisition of senescence related genes in UC and CRC

A comprehensive list of cellular senescence-associated genes was obtained from the CellAge database. By intersecting the gene sets from WGCNA modules, DEGs, and the CellAge dataset via “ggvenn” package (v 0.1.9) (Gao et al., 2024), we extracted a subset of genes that are not only involved in cellular senescence but also exhibit differential expression and co-expression patterns in UC and CRC. These intersecting genes were considered as potential senescence-related biomarkers and therapeutic targets for further analysis.

### 2.5 Gene set enrichment analysis on functions and pathways

Gene Ontology (GO) provides a structured, dynamically updated vocabulary encompassing gene product attributes across all species, in which GO enrichment contained 3 parts: biological processes (BPs), cellular components (CCs) and molecular functions (MFs) (Zhao et al., 2022). Kyoto Encyclopedia of Genes and Genomes (KEGG) integrates genomic, chemical, and systemic functional information, offering insights into the network of molecular interactions in the cells. For the purpose of understanding candidate genes’ function as well as participating pathways, “clusterProfiler” package (v 4.7.13) was employed for GO and KEGG analysis (Wu et al., 2021). Utilizing GO and KEGG pathway analyses, we systematically explore the functional and interactive networks that characterize the senescence landscape in UC transitioning into CRC.

### 2.6 Construction and validation of the integrated machine learning (IML) model

We developed the final predictive model with optimal performance by applying 10-fold cross-validation on the training set, evaluating 113 model combinations derived from 12 machine learning algorithms. These algorithms included Lasso, Ridge, Stepwise GLM (Stepglm), Random Forest (RF), XGBoost, Elastic Net (Enet), Linear Discriminant Analysis (LDA), Partial Least Squares Regression for Generalized Linear Models (plsRglm), Generalized Boosted Regression Models (GBM), Naive Bayes, GLMBoost, and Support Vector Machine (SVM). The 113 models consisted of 22 individual algorithms and 91 combined algorithms, as detailed in Supplementary Table S2. To determine the best-performing model, we calculated the concordance index (C-index) for each model and selected the one with the highest C-index as the optimal model. The genes identified by this model were considered candidate disease-related genes, potentially serving as biomarkers for UC and CRC.

After constructing the integrated machine learning (IML) model, we assessed its classification performance using confusion matrices for the training set and two independent validation datasets, GSE13367 and GSE90627. To further validate the model’s predictive capability, we generated Receiver Operating Characteristic (ROC) curves for both the training and validation sets and computed the Area Under the Curve (AUC) with 95% Confidence Intervals (CI). A model was deemed statistically rational only if the ROC AUC exceeded 0.7 for both the training and validation sets (Qin et al., 2023; Chen B. et al., 2024). This approach ensured the robustness and generalizability of our model in distinguishing disease-associated genes and validating their diagnostic potential.

### 2.7 Differential gene expression analysis and ROC curve construction

Differential gene expression analysis was performed using experimental data from the GEO datasets. To compare the expression levels of disease-related genes between the UC and CRC validation cohorts, we conducted Student’s t-test. Genes exhibiting statistically significant differential expression (p < 0.05) were identified as cellular senescence-related genes in UC or CRC. To evaluate their diagnostic potential, we generated Receiver Operating Characteristic (ROC) curves for each gene and calculated the Area Under the Curve (AUC) with 95% Confidence Intervals (CI). Genes with an AUC greater than 0.7 in both UC and CRC patients were considered to have significant diagnostic value (Liu et al., 2024). Furthermore, the significantly differentially expressed genes were integrated into a combined diagnostic model, and its ROC curve was constructed. If the combined model exhibited an AUC higher than that of any individual gene, it was considered a more effective diagnostic tool. The volcano plot was redrawn to visualize the upregulation or downregulation of genes with significant expression differences between UC and CRC.

### 2.8 Construction of the protein-protein interaction (PPI) network

A protein-protein interaction (PPI) network was constructed to explore the functional relationships and interaction dynamics among the genes with significant expression differences identified in IML. GeneMANIA (http://genemania.org/) incorporates data from multiple interaction types, including co-expression, physical interactions, genetic interactions, co-localization, pathway participation, and shared protein domains, providing a holistic view of the gene interactions. The genes with significant expression differences were input into GeneMANIA to generate a comprehensive PPI network.

### 2.9 Analysis for immune cell infiltration

To investigate disparities in immune infiltration between the two risk groups, the infiltration abundance of 22 distinct immune cell types (Chen et al., 2019) was first quantified using the CIBERSORT algorithm as implemented in the IOBR package (v 0.99.9) (Zhang et al., 2024). A Wilcoxon test was then applied to identify immune cell populations displaying significant differences (p < 0.05) between the risk groups. Subsequently, Spearman correlation analyses were performed with the psych package (v 2.4.3), using thresholds of |cor| > 0.3 and p < 0.05, to elucidate the correlation network among these differentially abundant immune cells. In addition, correlations between these immune cells and prognostic genes were evaluated under the same thresholds to further characterize the interplay between immune infiltration and gene expression profiles.

### 2.10 Analysis of gene expression and microsatellite instability across tumor stages in COAD

We conducted a comprehensive analysis of the expression patterns of five candidate genes (ABCB1, CXCL1, TACC3, TGFBI, and VDR) in colorectal adenocarcinoma (COAD) using publicly available RNA sequencing data from The Cancer Genome Atlas (TCGA) database. Microsatellite instability (MSI), a marker indicative of genomic instability and immunogenicity in colorectal cancer, was evaluated by computing Pearson correlation coefficients between gene expression and MSI scores. These correlations, along with significance levels (p-values), were visualized using radar charts, providing intuitive insights into the association between gene expression and MSI status (Lin et al., 2020). To examine potential differences in gene expression across tumor stages (Stages I, II, III, and IV), expression levels were visualized through boxplots, with statistical significance between stage groups determined via Wilcoxon rank-sum tests, a non-parametric method suitable for small or unevenly distributed clinical cohorts (Liu et al., 2023).

### 2.11 Identification of novel drug targets

To explore potential therapeutic agents targeting cellular senescence-related genes in UC and CRC, we conducted a comprehensive drug enrichment analysis using Enrichr (https://maayanlab.cloud/Enrichr/). Initially, significant candidate compounds were screened with strict criteria, specifically applying thresholds of p-value <0.05 to ensure statistical robustness. Subsequently, we performed molecular docking analysis to validate and refine the candidate selection using the CB-Dock2 platform, an advanced version of the CB-Dock server optimized for protein-ligand blind docking. CB-Dock2 integrates cavity detection, molecular docking, and homologous template fitting to provide precise predictions of binding sites and affinities between proteins and ligands (https://cadd.labshare.cn/cb-dock2/index.php).

The selection of final candidate drugs followed a clearly defined, stepwise filtering process. Initial drug enrichment analysis identified compounds significantly interacting with the target genes. Candidate drugs passing enrichment thresholds with the lowest p-value underwent molecular docking analyses. Docking scores (Vina scores) obtained from CB-Dock2 were employed, with lower scores indicating stronger binding affinity and better potential therapeutic efficacy. Compounds with the lowest Vina scores were prioritized as potential therapeutic candidates based on their binding strength and interaction specificity, then we used CB-Dock to visualize the docking result.

Through this combined approach of enrichment analysis and molecular docking, we systematically and rigorously identified promising candidate drugs, thereby enhancing the potential for targeted therapeutic strategies in UC and CRC.

## 3 Results

### 3.1 Acquisition of senescence related genes in UC and CRC

**FIGURE 2:**
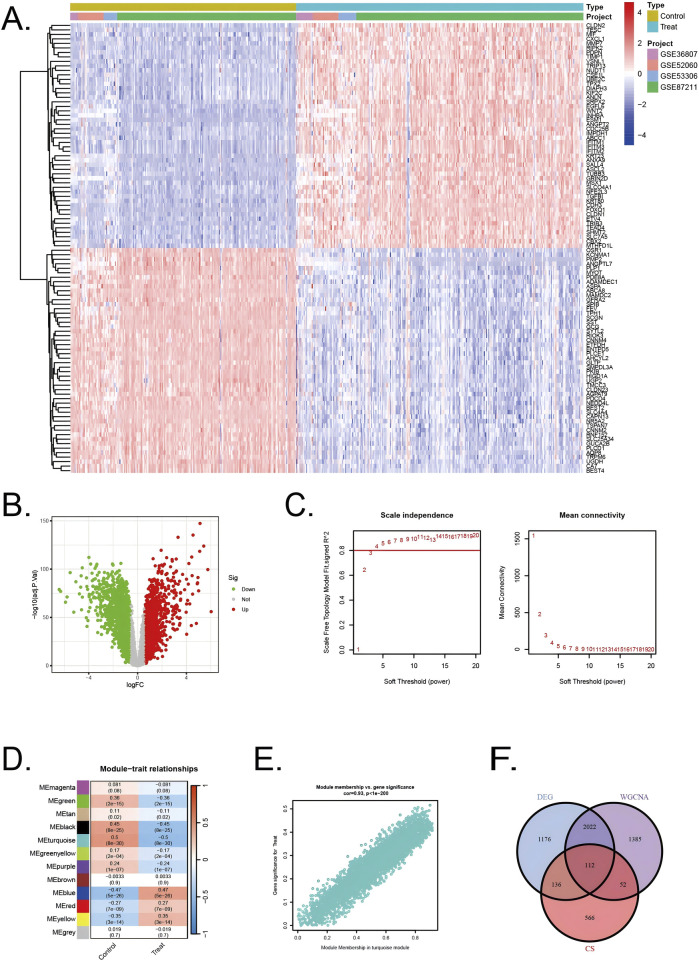
Identification of DEGs in UC/CRC patients and identification of key genes by WGCNA analysis in UC/CRC patients **(A)** Heatmap showing upregulated or downregulated DEGs in UC/CRC samples compared to normal samples (bule: downregulated; red: upregulated) **(B)** Volcano plot of DEGs between UC/CRC and controls. **(C)** Analysis of network topology for various soft thresholds (β) **(D)** Module-trait relationships. **(E)** Associations between turquoise module membership and gene importance is depicted in a scatter plot. **(F)** The overlapping regions from key module genes, DEGs, and cellular senescence related genes.

All diseased samples from the training set (GSE52060, GSE87211, GSE36807, and GSE53306) were merged into “Treat”, and all healthy control samples were merged into “Control”. In the heatmap, the validation group and experimental group are divided into different modules, and the samples in each dataset are segmented into different squares (Figure 2A). The colors of the squares represent the changes in gene expression, with red representing upregulation and blue representing downregulation. A total of 3,446 DEGs were identified by comparing the BD and control groups. Among all DEGs, 1716 genes displayed upregulation, whereas 1730 genes were downregulated (Figure 2B).

Next, WGCNA was used to identify the significant module genes associated with UC and CRC. We selected the optimal soft-thresholding power (β) to establish a scale-free topology network, ensuring that the scale-free topology fit index (R^2^) exceeded 0.8 (Figure 2C). The chosen β value was set to maintain the network’s scale-free characteristics. The grey module and brown module did not successfully cluster the genes commonly considered irrelevant or uninformative (i.e., the “junk module”). The turquiose (r = 0.5, p = 8 × 10^−30^) module displayed the highest correlation with UC and CRC (Figure 2D). The relationship between module membership and gene significance in the turquiose module is calculated (Cor = 0.93, p < 10^–200^) and plotted (Figure 2E). Consequently, 3,571 significant module genes were identified.

Through a comprehensive analysis integrating data from the CellAge database, weighted gene co-expression network analysis (WGCNA) modules, and differentially expressed genes (DEGs), we identified a refined subset of 112 shared genes implicated in cellular senescence and their association with UC and CRC. The Venn diagram (Figure 2F) summarizes the intersection results, illustrating the overlap between the gene sets and emphasizing the genes that could serve as pivotal links between cellular senescence and disease progression in UC and CRC.

### 3.2 Functional annotation and pathway enrichment analysis

**FIGURE 3:**
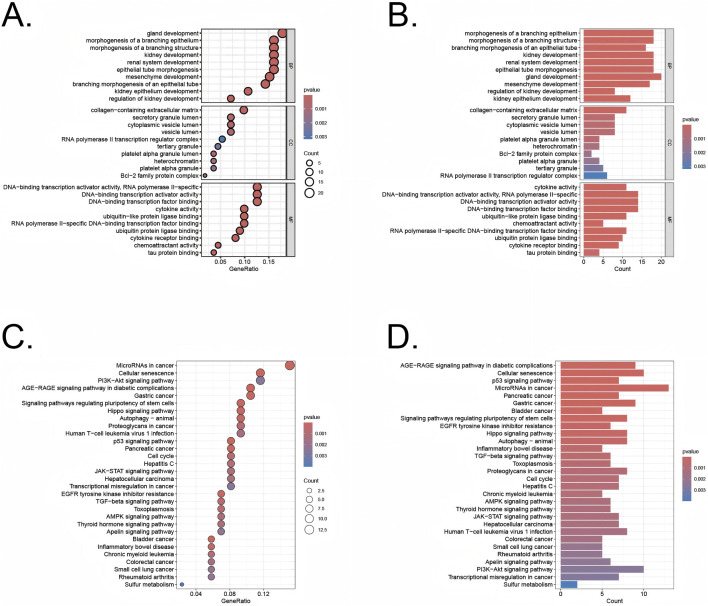
GO and KEGG analysis of the overlapping genes **(A,B)** GO analysis of these overlapping genes in UC/CRC patients. **(C,D)** KEGG analysis of these overlapping genes in UC/CRC patients.

The differentially expressed genes identified using the limma R package were analyzed through Gene Ontology (GO) enrichment analysis (Figures 3A,B). The results were ranked in ascending order based on adjusted p-values (p.adjust) and GeneRatio. In the Biological Processes (BP) category, the top three pathways with the lowest p.adjust values and the highest number of enriched genes were morphogenesis of a branching structure, morphogenesis of a branching epithelium, and gland development. In the cellular components (CC) category, the top 3 terms were cytoplasmic vesicle lumen, secretory granule lumen, and collagen−containing extracellular matrix. In the molecular functions (MF) category, the top 3 terms were DNA−binding transcription factor binding, DNA−binding transcription activator activity, DNA−binding transcription activator activity, RNA polymerase II−specific. The KEGG pathway analysis revealed key pathways that were significantly enriched among the genes identified in our study (Figures 3C,D). These pathways included the PI3K-Akt signaling pathway, p53 signaling pathway, and the cell cycle, which are known to play pivotal roles in regulating cellular senescence, survival, proliferation, and apoptosis. The involvement of these pathways underscores the potential mechanisms through which cellular senescence could influence the transition from UC to CRC.

### 3.3 Identification of intersection genes with diagnostic value and developing a diagnostic model for UC-related CRC via machine learning

**FIGURE 4:**
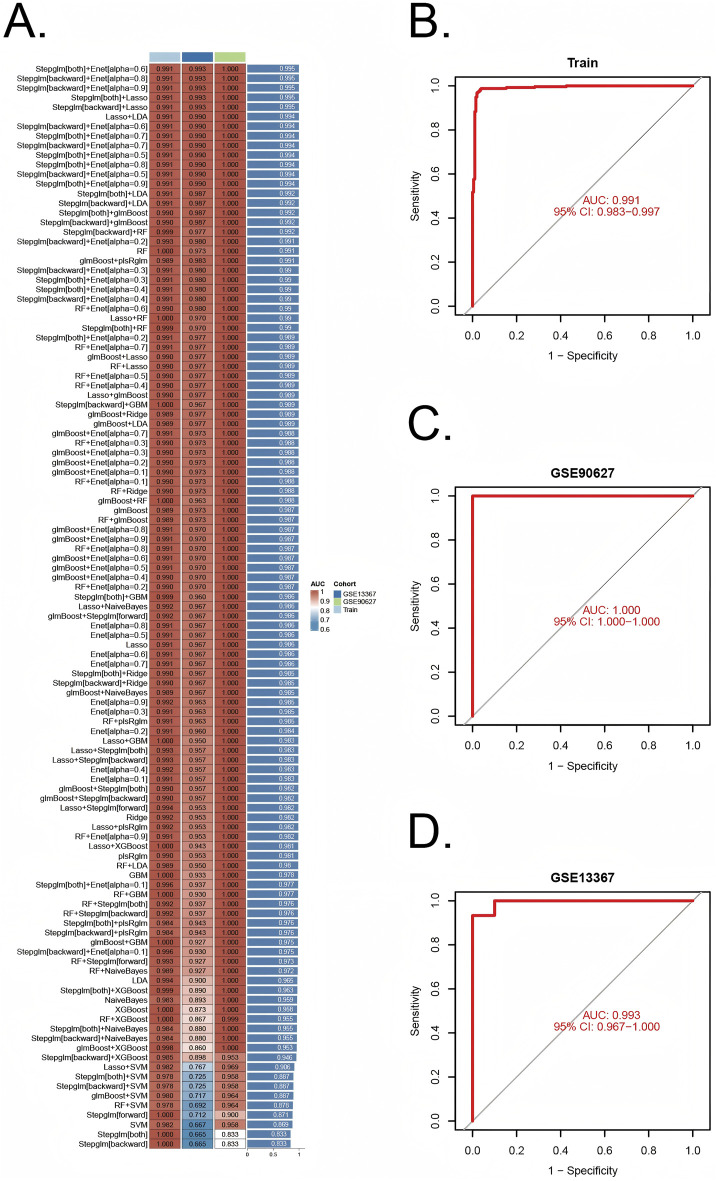
Construction and validation of diagnostic signatures by integrative machine learning **(A)** The 113 combinations of prediction models using 10-fold cross-validation with ranked AUC index. **(B–D)** ROC plots for datasets in internal training set and external validation sets (GSE90672 and GSE13367), correspondingly.

A comprehensive machine learning approach involving 12 algorithms was implemented with a 10-fold cross-validation process to identify the most robust diagnostic model based on 112 shared genes (Figure 4A). The analysis was conducted using the training dataset and validated across two external datasets (GSE90672 and GSE13367). The final model, which demonstrated the best performance, was constructed by integrating Stepglm [both] and Enet [α = 0.6]. Specifically, the Stepglm [both] algorithm identified 10 pivotal genes, including ABCB1, AGR2, BCL2L1, CXCL1, FOXO1, SOX4, TACC3, TGFβI, VDR, and VEGFA, while the Enet [α = 0.6] algorithm optimized the model’s reliability. The validation datasets remained completely independent and were not involved at any stage of feature selection, model training, parameter tuning, or optimization, thereby preventing any potential data leakage or information contamination. Furthermore, all cross-validation procedures, feature selection steps, and modeling approaches were performed exclusively within the training set. The calibration curves, illustrated in Figures 6D,E, show high AUC values for the training set (Figure 4B, AUC = 0.991), as well as the testing set GSE90627 (Figure 4C, AUC = 1.000) and GSE13367 (Figure 4D, AUC = 0.993), indicating a strong agreement between the predicted probabilities and observed clinical outcomes. These results highlight the robust calibration and diagnostic performance of the proposed model.

### 3.4 Diagnosis value of pivotal genes

**FIGURE 5:**
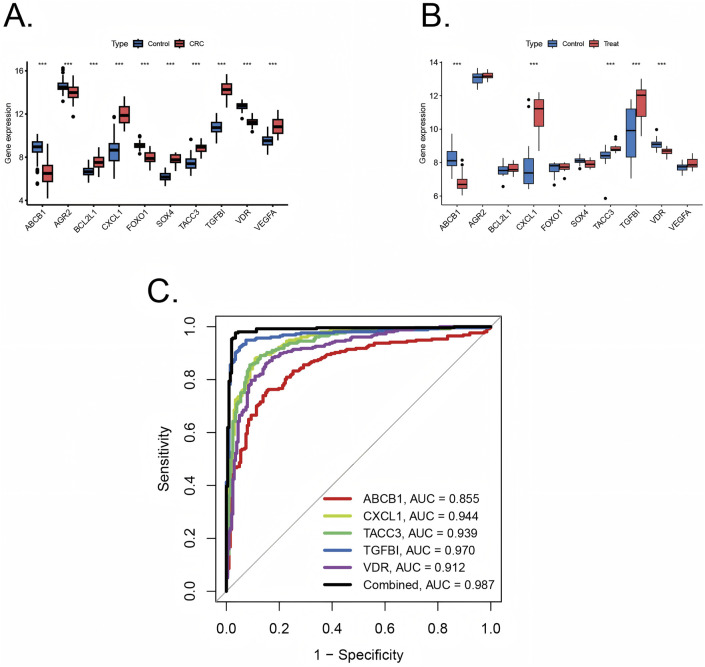
Validation of diagnostic value of pivotal genes **(A)** Pivotal genes expression in colorectal cancer training sets with significance (***p < 0.001) **(B)** Pivotal genes expression in ulcerative colitis training sets with significance (***p < 0.001). **(C)** ROC plots for each diagnostic gene and the combined model in internal training cohorts.

Ten pivotal genes were included in the following ROC analysis. All 10 pivotal genes showed high significance (p < 0.001) in CRC and its control group (Figure 5A), while ABCB1, CXCL1, TACC3, TGFβI, VDR displayed high significance (p < 0.001) in UC and its control group (Figure 5B). Based on the significant differences in gene expression, ABCB1, CXCL1, TACC3, TGFβI, and VDR were integrated into a combined model. All the 5 genes were included in ROC analysis. We calculated the AUC values for each gene and the combined model separately (Figure 5C). The results showed that the AUC values of all genes were not less than 0.7, and the AUC value of the combined model (AUC = 0.989) was higher than that of any individual gene. Therefore, the combined model has greater diagnostic value compared to any individual gene.

### 3.5 Protein-protein interaction (PPI) network construction

**FIGURE 6:**
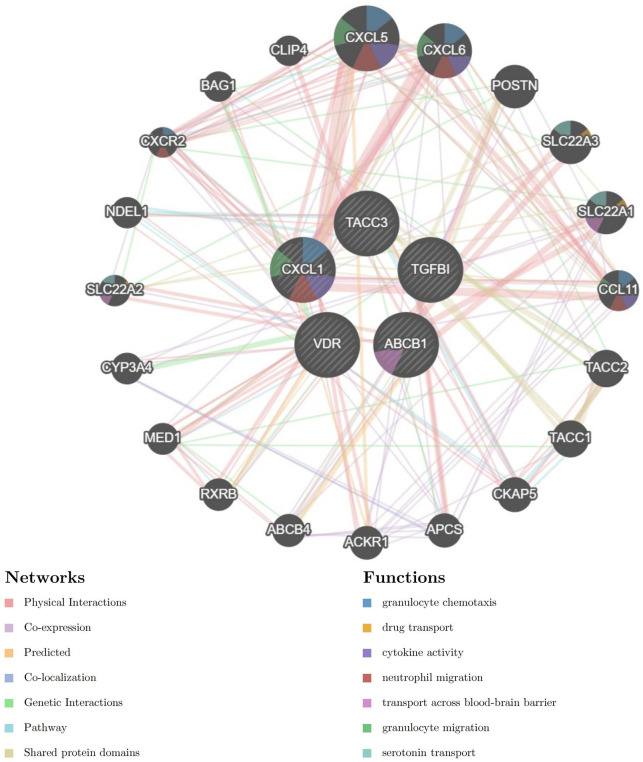
Protein-Protein Interaction (PPI) network for five combined model genes and their related biological functions.

The PPI network for genes which were included in combined model genes (ABCB1, CXCL1, TACC3, TGFβI, and VDR) were created via the GeneMANIA database (https://genemania.org/). In the GeneMANIA map, a total of 20 genes (CXCL5, CXCL6, POSTN,SLC22A3, SLC22A1, CCL11, TACC2, TACC1, CKAP5, APCS, ACKR1, ACBC4, RXRB, MEDI, CYP3A4, SLC22A2, NDEL1, CSCR2, BAG1, and CLIP4) were found to have gene interactions with five combined model genes (Figure 6). In the GeneMANIA network, physical interactions between pivotal genes and other genes account for 77.64%, while co-expression accounts for 8.01%, demonstrating the strong protein-protein interactions within the GeneMANIA network topology.

### 3.6 Analysis of immuno-infiltration and correlation analysis

**FIGURE 7:**
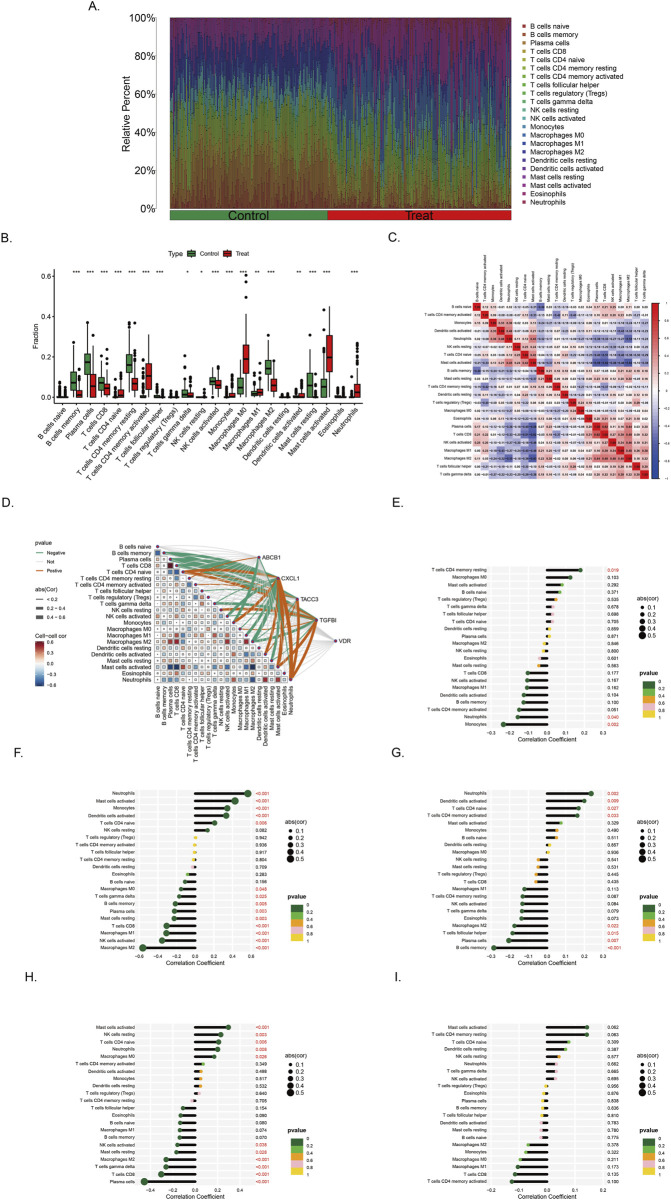
Immune infiltration landscape in colorectal cancer and ulcerative colitis **(A)** Proportional graph of 22 kinds immune cells in all training sets. **(B)** Distribution of different types of immune cells in control group and CRC/UC group (*p < 0.05, **p < 0.01, ***p < 0.001) **(C)** Correlation of 22 immune cells by compositions. Both horizontal and vertical axes demonstrate immune cells subtypes. **(D)** Correlation analysis of the level of infiltration of five pivotal genes and each type of immune cells. **(E–I)** The association between ABCB1, CXCL1, TACC3, TGFβI and VDR expression with different immune cell infiltration in the treat group, correspondingly.

Immune correlation analysis was performed with all samples in training set (Figure 7A). The infiltration landscape showed that 22 kinds of immune cell distributions in the control and treat groups. Fourteen types of immune cells (neutrophils, mast cells activated, mast cells resting, macrophages M2, macrophages M0, monocytes, NK cells activated, T cells follicular helper, T cells CD4 memory activated, T cells CD4 memory resting, T cells CD4 naive, T cells CD8, and B cells memory) infiltrated significantly (p < 0.001) between the control and treat groups (Figure 7B). Correlation analysis between immune cells indicates that Macrophage M2 exhibited significantly negative correlation with activated T cells CD4 naive (r = −0.63, p < 0.05), T cells CD8 had positive correlation with macrophages M2 (r = 0.31, p < 0.05) (Figure 7C).

The correlation between genes and 22 immune cell types, as well as the interrelationships among immune cells, has been systematically analyzed and visualized (Figure 7D). Among the findings, ABCB1 exhibits the strongest positive correlation with T cells CD4 memory resting, while demonstrating the most pronounced negative correlation with Monocytes (Figure 7E). Similarly, CXCL1 is most positively correlated with Neutrophils and Macrophages M0, whereas its most significant negative associations are observed with NK cells activated and Macrophages M2 (Figure 7F). In the case of TACC3, its highest positive correlation is identified with Neutrophils, whereas its strongest negative correlations are noted with B cells memory and Plasma cells (Figure 7G). Notably, TGFβI shows the most significant positive correlation with Mast cells activated, while displaying a marked negative correlation with Plasma cells, T cells CD8, T cells gamma delta, and Macrophages M2 (Figure 7H). The gene VDR did not exhibit strong correlations with immune cells in the analysis (Figure 7I).

### 3.7 Stage-dependent expression and MSI correlation of ABCB1, CXCL1, TACC3, TGFBI, and VDR in COAD

**FIGURE 8:**
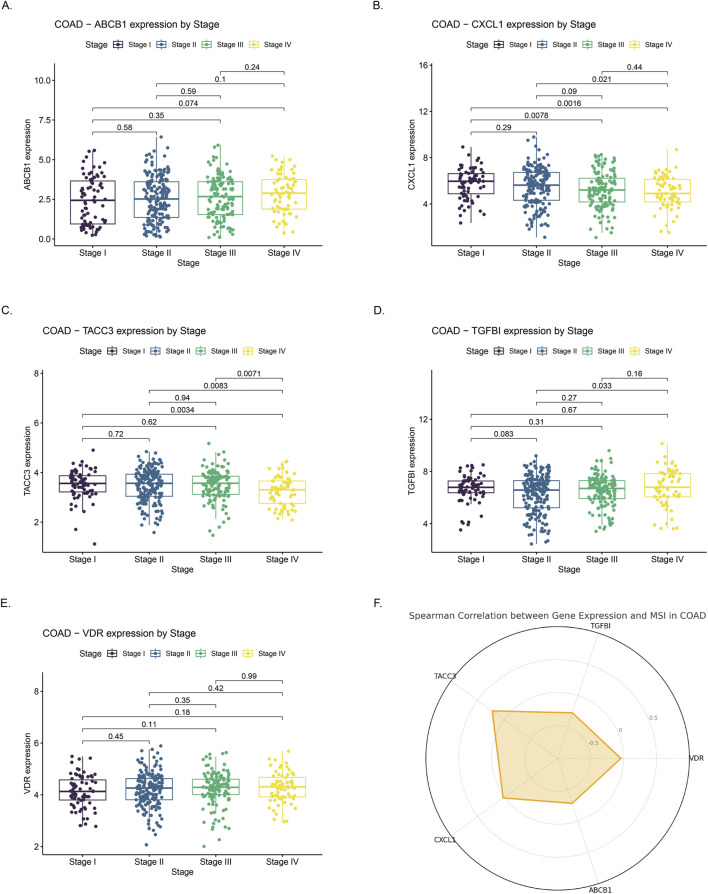
Correlation of gene expression with clinical stage and microsatellite instability (MSI) status in colon adenocarcinoma (COAD) **(A–E)** Expression profiles of ABCB1, CXCL1, TACC3, TGFBI, and VDR across COAD stages I to IV. Significant differences in expression were observed across stages. **(F)** Radar chart illustrating Spearman correlation coefficients between expression levels of the five genes and MSI status in COAD. Positive correlations suggest higher gene expression is associated with increased MSI, whereas negative correlations indicate an inverse relationship.

ABCB1 expression significantly decreased with advancing tumor stage, with higher median expression observed in early-stage (Stage I/II) compared to late-stage tumors (Stage III/IV), consistent with earlier reports of its downregulation in colorectal carcinogenesis (Figure 8A). Conversely, CXCL1 and TACC3 were progressively upregulated in advanced stages. CXCL1 showed significantly elevated expression in Stage III–IV tumors compared to Stage I–II, indicative of enhanced inflammation (Figure 8B). Similarly, TACC3 levels significantly increased in late-stage disease, aligning with its previously reported association with tumor progression and poor prognosis in colorectal cancer (Figure 8C). TGFBI and VDR exhibited no clear stage-dependent expression patterns. TGFBI expression fluctuated without significant differences, while VDR levels remained relatively stable across stages, aligning with previous studies reporting limited stage-dependent variation (Figures 8D,E). MSI analysis revealed significant positive correlations for TACC3 and CXCL1 (p < 0.05), suggesting their potential roles in MSI-high tumor biology, whereas ABCB1, TGFBI, and VDR lacked significant associations with MSI status (Figure 8F).

### 3.8 Potential drug discovery and gene-drug interaction

**FIGURE 9:**
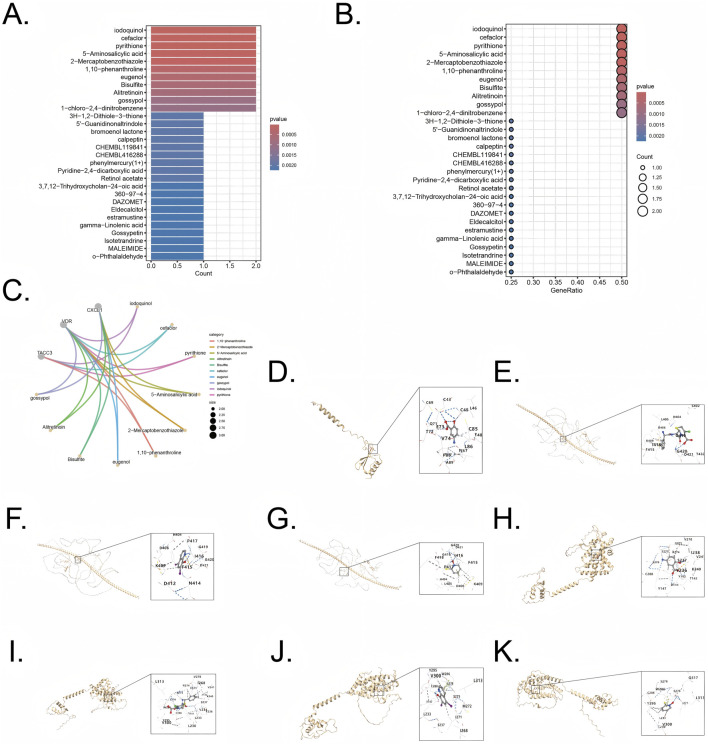
Drug enrichment and molecular docking for five combined model genes **(A,B)** Exploration potential drug from Enrichr to five pivotal genes. **(C)** Visualization of chemical compound data illustrating the distribution and categorization of various pharmaceutical agents **(D)** Visualization of molecular docking for 5-Aminosalicylic acid to its target CXCL1. **(E)** Visualization of molecular docking for cefaclor to its target TACC3 **(F)** Visualization of molecular docking for iodoquinol to its target TACC3. **(G)** Visualization of molecular docking for pyrithione to its target TACC3 **(H)** Visualization of molecular docking for 5-Aminosalicylic acid to its target VDR. **(I)** Visualization of molecular docking for cefaclor to its target VDR **(J)** Visualization of molecular docking for iodoquinol to its target VDR. **(K)** Visualization of molecular docking for pyrithione to its target VDR.

Enrichr database was utilized to screen therapeutic agents targeting the five combined model genes. The analysis highlighted several compounds potentially effective in targeting genes associated with UC and CRC, and the compounds (p < 0.05) are listed in Supplementary Table S2. Protein sequences were obtained from Uniprot (https://www.uniprot.org/), and compound structures were retrieved from PubChem (https://pubchem.ncbi.nlm.nih.gov/). The identified therapeutic agents included iodoquinol, cefaclor, pyrithione, 5-Aminosalicylic acid, 2-Mercaptobenzothiazole, 1,10-Phenanthroline, eugenol, Bisulfite, Alitretinoin, and gossypol (Figures 9A,B).

Genes CXCL1 (Uniprot ID: P09341), TACC3 (Uniprot ID: Q9Y6A5), and VDR (Uniprot ID: P11473) were selected as target proteins, and their interactions with candidate drugs were visualized through interconnected curves, enabling clearer exploration of drug-target relationships (Figure 9C). The four most significant drugs, iodoquinol (CID: 3,728), cefaclor (CID: 51,039), pyrithione (CID: 1570), and 5-Aminosalicylic acid (CID: 4075), were chosen based on the lowest enrichment p-values for further validation using molecular docking. Docking results, represented by Vina scores, are summarized in Table 1. Lower Vina scores indicate stronger binding affinities, justifying the prioritization of these compounds as therapeutic candidates. Visualization of docking interactions was performed using PyMOL software (Figures 9D–K). Importantly, 5-Aminosalicylic acid is already clinically established for UC treatment, providing additional validation and supporting the overall reliability and validity of our model.

**TABLE 1:** Binding affinities (Best Vina Scores) of candidate drugs with target proteins.

Protein-drug pair	Best vina score
CXCL1-5-Aminosalicylic acid	−4.7
TACC3-cefaclor	−6.4
TACC3-iodoquinol	−4.9
TACC3-pyrithione	−3.9
VDR-5-Aminosalicylic acid	−6.0
VDR-cefaclor	−9.7
VDR-iodoquinol	−5.6
VDR-pyrithione	−4.7

## 4 Discussion

Sustained ulcerative colitis of the colorectal leads to tissue damage and repair, which is associated with an increased incidence of colitis-associated colorectal cancer. Meanwhile, cellular senescence may be a trigger for colorectal cancer or an emerging therapeutic target (Wu et al., 2022). To our knowledge, our work is the first to filter senescence-related genes and potential therapeutic drugs in UC and CRC based on the overall normalized weights of IML. Four training sets, two testing set, a total of 621 samples in GEO database were included, and clinical studies were promoted by using datasets to validate the results. Five genes in combined model, ABCB1, CXCL1, TACC3, TGFβI, and VDR, all showed an AUC >0.7 in gene ROC plot, and their combination diagnostic model showed higher AUC value than any other individual genes, indicating a potential diagnostic value of the five combined model genes. We further investigated the immune correlations of the five genes in the combined model and expanded their potential diagnostic value. These genes highlight the intricate relationship between cellular senescence, immune response, and tumor progression in CRC. Moreover, the development of novel anti-cancer, anti-inflammatory, and anti-aging drugs is often costly and time-consuming. By leveraging bioinformatics to identify medications targeting these key genes, our approach has the potential to enhance efficiency and significantly reduce the costs associated with drug discovery.

ABCB1, also known as P-glycoprotein (P-gp) or MDR1, is a type of ATP-binding cassette (ABC) transporter. The gene encodes a membrane-bound protein that belongs to the ATP-binding cassette (ABC) transporter superfamily. This protein functions as an ATP-driven drug efflux pump, capable of exporting a wide range of xenobiotic compounds due to its broad substrate specificity. ABCB1 helps protect cells from toxic compounds but also contributes to multidrug resistance (MDR) in colorectal cancer cells by reducing the intracellular concentration of anticancer drugs, including doxorubicin, paclitaxel, and vincristine, making them less effective (Tan et al., 2022; Lei et al., 2024). In UC, the dysfunction or low activity of ABCB1 leads to the accumulation of harmful bacterial products within the gut epithelium, contributing to chronic inflammation and mucosal damage. This impaired function disrupts the balance of the gut microbiome, exacerbating the inflammatory response and promoting the development of UC symptoms (Stoeltje et al., 2024). Furthermore, ABCB1 has been identified as a cell senescence related gene, the expression of ABCB1 may increase during aging to enhance the resistance of cells to external toxic substances (Wu et al., 2023). ABCB1 can also alter how cells respond to stress or therapy by shifting cell fate from apoptosis toward survival outcomes like senescence. For example, cancer cells overexpressing P-gp are less prone to undergo apoptosis after DNA damage (e.g., radiation) and instead exhibit higher incidences of senescence and mitotic catastrophe (Tainton et al., 2004).

CXCL1 is a potent neutrophil chemoattractant that plays a significant role in the immune response. CXCL1’s function in UC is to facilitate the migration and activation of immune cells, thereby exacerbating the inflammatory response in the colon, which is positively correlated with UC severity (Huo and Wang, 2023). CXCL1 plays a significant role in CRC by promoting tumor progression through several mechanisms. It is overexpressed in colorectal cancer tissues and contributes to cancer cell proliferation, migration, and invasion. CXCL1 activates the NF-κB pathway, which is crucial for cancer cell survival and inflammation (Zhuo et al., 2022). Additionally, CXCL1 recruits myeloid-derived suppressor cells (MDSCs) via the CXCL1-CXCR2 axis, which helps the tumor evade the immune system. CXCL1 is part of the senescence-associated secretory phenotype (SASP), involves its role in the tumor microenvironment, and helps wake up dormant cancer cells, making them more aggressive and prone to recurrence (Korbecki et al., 2022). Thus, CXCL1 is a significant SASP component mechanistically linked to senescence: it reinforces senescence via autocrine signaling, and its presence in the secretome can modulate immune surveillance and tissue outcomes in age-related pathologies (Chambers et al., 2021).

TACC3, a member of the transforming acidic colied-coil protein family, is found to be overexpressed in colorectal cancer tissues, contributing to increased cell proliferation and cellular senescence. TACC3 regulates various processes during mitosis and interphase. During mitosis, it interacts with proteins like KIFC1 to cluster extra centrosomes, preventing multipolar spindle formation and ensuring proper cell division (Saatci et al., 2023). In interphase, TACC3 interacts with the NuRD complex to suppress tumor suppressor genes, promoting cell cycle progression and survival (Saatci et al., 2023). Transcriptomic analyses of colonic tissues identified TACC3 as significantly upregulated in UC patients, ranking it among a handful of pivotal genes distinguishing UC from healthy tissue. Elevated TACC3 expression in the inflamed colonic mucosa may reflect increased epithelial cell proliferation and altered regenerative responses during chronic inflammation (Zeng et al., 2018). Targeting TACC3 with inhibitors can induce mitotic catastrophe and G1 phase arrest, leading to cancer cell death, making it a promising therapeutic target for aggressive cancers. Additionally, high TACC3 expression is linked to an immunosuppressive tumor microenvironment and higher tumor mutational burden, suggesting its involvement in tumor progression and immune evasion. Knockdown of TACC3 reduces cell proliferation and senescence, indicating its potential as a therapeutic target (Du et al., 2016).

TGFβI, or Trnsforming Growth Factor Beta-Induced protein, is a RGD-containing protein that binds to type I, II and IV collagens, playing a significant role in cancer, particularly in colorectal cancer (CRC) (Chiavarina et al., 2021). It is involved in promoting angiogenesis, which is the formation of new blood vessels, thereby supporting tumor growth and metastasis. TGFβI’s presence pushed cells into cellular senescence (with even telomerase activity paradoxically rising as often seen in stress-induced senescence), and conversely, loss of TGFBI was one factor allowing those cancer cells to escape senescence and continue dividing (Li et al., 2012). In UC, TGFBI expression is elevated in the inflamed colon, indicating activation of wound-healing and fibrotic pathways in the mucosa (Haberman et al., 2020). TGFβI expression is regulated by TGFβ signaling pathways, and its presence is associated with increased metastatic potential in CRC cells. TGFβI’s interactions with extracellular matrix proteins and integrins are crucial for its role in cancer, influencing cell adhesion, migration, and chemotherapy resistance (Corona and Blobe, 2021). TGFβI downstream gene TGF-β1 is a key cytokine involved in the development of kidney diseases and can induce the expression of p21, a protein that can regulate cell cycle arrest and senescence (Ueda et al., 2021).

VDR is a nuclear hormone receptor for 1,25-dihydroxyvitamin D_3_ that plays a multifaceted role in cellular senescence and aging. In general, active vitamin D/VDR signaling has anti-senescent and pro-homeostatic effects in cells. Vitamin D can attenuate oxidative stress and delay the onset of senescence largely by inducing antioxidant and longevity genes–for example, VDR activation elevates Nrf2 (a master regulator of antioxidant response) and Klotho (an anti-aging protein), which improves mitochondrial function and reduces reactive oxygen species (Chen J. et al., 2024). VDR plays a crucial role in both colorectal cancer (CRC) and ulcerative colitis (UC). In CRC, VDR helps regulate the immune response and inflammation, which are key factors in cancer progression. VDR deficiency is linked to more severe colitis and an increased risk of developing colorectal cancer. It modulates macrophage polarization, promoting an anti-tumor M1 phenotype over the pro-tumor M2 phenotype (Hu et al., 2020). This regulation helps prevent the transition from chronic colitis to colorectal cancer. In UC, VDR’s role is similar, as it helps control inflammation and maintain intestinal barrier integrity, reducing the risk of cancer development (Shi et al., 2020). M1 macrophages have anti-tumor functions, which help in reducing inflammation and preventing the progression of colitis-associated colorectal cancer. The absence of VDR accelerates the progression from chronic colitis to colorectal cancer, highlighting its protective role in this transition. VDR plays a crucial role in regulating DNA repair during oncogene-induced senescence (OIS) (Graziano et al., 2016). When VDR levels are reduced, as seen in cells expressing oncogenic Ras, it leads to a decrease in the DNA repair factors BRCA1 and 53BP1. This reduction impairs the cell’s ability to repair DNA damage, contributing to genomic instability. VDR helps maintain the balance of these repair factors, and its downregulation can exacerbate DNA repair deficiencies, promoting senescence and potentially leading to tumorigenesis (Graziano et al., 2016).

Our Immuno-infiltration revealed that chronic inflammation in UC creates a pro-tumorigenic microenvironment that drives the inflammation-dysplasia-carcinoma sequence. UC-affected colonic tissue is heavily infiltrated by neutrophils, monocytes/M0 macrophages, and activated T cells, which sustain mucosal injury and promote regenerative proliferation (Penrose et al., 2021). These cells release inflammatory cytokines, chemokines, and reactive oxygen species that induce epithelial DNA damage and activate tumorigenic pathways. Neutrophils, for example, release myeloperoxidase and other mediators that exacerbate tissue damage and genomic instability, and their accumulation correlates with increased cancer risk (Zhang C. et al., 2023). M1-polarized macrophages in UC produce TNF-α, IL-1β, and IL-6, activating NF-κB and STAT3 pathways that support epithelial hyperplasia and survival. As malignancy develops, the immune infiltrate shifts: macrophages adopt an M2 phenotype, secreting immunosuppressive (IL-10, TGF-β) and pro-angiogenic factors, while CD4+T cells transition from a Th1/Th17 to a Th2-dominant profile. Th2 cytokines (e.g., IL-4, IL-13) can directly promote DNA damage and mutation in epithelial cells (Wang et al., 2015). Meanwhile, regulatory T cells expand in CRC and suppress cytotoxic responses via IL-10 and TGF-β, creating an immune-tolerant environment. Additional contributors include mast cells, which release mediators that disrupt the extracellular matrix and promote vascular remodeling, and monocytes, which differentiate into immunosuppressive tumor-associated macrophages and MDSCs. NK cell activity may also be impaired in chronic inflammation, reducing their tumor surveillance capability. Together, these changes illustrate how persistent immune dysregulation in UC not only sustains inflammation but also drives the molecular and cellular events underlying malignant transformation and immune escape in CRC (Li et al., 2023). In summary, the altered immune cell landscape in UC not only perpetuates inflammation but also initiates oncogenic changes, and as UC progresses to CRC, the immune contexture increasingly favors tumor progression (via growth and angiogenesis signals) and immune escape, mechanistically linking chronic colitis to colorectal carcinogenesis.

In comparison to previous UC and CRC gene signature studies (Chen et al., 2021; Shi et al., 2024; Huang et al., 2022; Horaira et al., 2023; Chadha, 2025), which predominantly uncovered overlapping inflammation- and immune-related biomarkers (e.g., IL1B, CXCL10) through standard differential expression or network analyses, our study takes a fundamentally different approach. We employed a senescence-based gene selection strategy, focusing on genes linked to cellular aging processes in the colitic mucosa, an aspect largely overlooked in earlier work. This novel focus yielded a distinct panel of senescence-associated genes (including ABCB1, CXCL1, TACC3, TGFBI, and VDR) that demonstrated superior diagnostic performance. In fact, our gene set achieved markedly higher accuracy in distinguishing disease states on independent validation cohorts (GSE13367 and GSE90627) than the signatures reported in prior studies. Mechanistically, our findings highlight cellular senescence as a key link between chronic inflammation and neoplastic transformation in ulcerative colitis, providing insights that earlier immune-centric signatures did not. Although CXCL1 has been reported, our research has important therapeutic implications: by pinpointing senescence drivers of colitis-associated carcinogenesis, and our study opens up new avenues for intervention (for example, targeting senescent cells or their secretory factors) to potentially prevent or delay UC progression to CRC, a clear advantage over previous gene sets that mostly served as diagnostic markers and revealing actionable pathogenic processes.

## 5 Conclusion

In this work, we have effectively applied integrative machine learning and bioinformatics approaches to identify key cellular senescence-related genes, namely, ABCB1, CXCL1, TACC3, TGFβI, and VDR, that show promising potential as diagnostic biomarkers and therapeutic targets in the progression from ulcerative colitis to colorectal cancer. Our combined diagnostic model, which outperformed individual gene markers, underscores the significant diagnostic value of these candidates, while our immune infiltration analyses further suggest that immunological dysregulation may play a crucial role in disease evolution. However, the current findings are primarily based on retrospective dataset analyses and predictive modeling, and thus additional experimental and clinical validations are required to fully ascertain the clinical applicability of these genes. In the future, we plan to expand our study with larger, diverse clinical cohorts and mechanistic investigations to further elucidate the roles of these senescence-related genes in UC and CRC, ultimately paving the way for more targeted and effective therapeutic strategies.

## Data Availability

The original contributions presented in the study are included in the article and Zenodo link: https://zenodo.org/records/17274390?token=eyJhbGciOiJIUzUxMiJ9.eyJpZCI6IjgzMjQ3ZDc3LTljYjMtNGVlYy1iYTI1LWRmM2MzYmVlM2M2YyIsImRhdGEiOnt9LCJyYW5kb20iOiIzNzU0Y2RjMDdmZTlkYzNmMWVmNTJjNTc5NTc4MTZkZiJ9.YAu5Lc4IMsUnTDAfdiiaX0cEFO66kH3-Tc0IBYMtxRf6O824e85uRvD_m2Dip9cDC_IUg2CIxisSaN5iwdyrFQ and https://zenodo.org/records/17274398?token=eyJhbGciOiJIUzUxMiJ9.eyJpZCI6ImRhZWM4ZDMzLWYxNTAtNGM2Ni04ZGQzLWM0YTQ4NmI4YjllOCIsImRhdGEiOnt9LCJyYW5kb20iOiI4OWE2MGMyMjFmYzFhMzBiZjYxNTJkYTMwYjQxZGJmOSJ9.22PVvcpoKy7oAhugaqx5iSgAL2v0DMbGO-obROSh16bxkeHw-eLIbeIfibTVjZ_1klseB8EH2NhfVPCAbyMygA. Further inquiries can be directed to the corresponding author.
