# Correction: Integrative machine learning and bioinformatics analysis to identify cellular senescence-related genes and potential therapeutic targets in ulcerative colitis and colorectal cancer

**DOI:** 10.3389/fbinf.2025.1750582

**Published:** 2025-12-03

**Authors:** Tianle Xue, Yunpeng Chen, Xiaomeng Li, Zhixiang Zhou, Qiyang Chen

**Affiliations:** 1 Strathclyde Institute of Pharmacy and Biomedical Sciences, University of Strathclyde, Glasgow, United Kingdom; 2 China Pharmaceutical University, Nanjing, China

**Keywords:** cellular senescence, ulcerative colitis, colorectal cancer, integrative machine learning, immune infiltration, therapeutic targets

**Affiliation** “China Pharmaceutical University, Nanjing, China” was omitted for author Tianle Xue, Yunpeng Chen, Xiaomeng Li, Zhixiang Zhou, and Qiyang Chen. This affiliation has now been added for all authors.

The **Data Availability statement** was erroneously given as “The datasets presented in this study can be found in online repositories. The names of the repository/repositories and accession number(s) can be found in the article/Supplementary Material.” The correct Data Availability statement is “The original contributions presented in the study are included in the article and Zenodo link: https://zenodo.org/records/17274390?token=eyJhbGciOiJIUzUxMiJ9.eyJpZCI6IjgzMjQ3ZDc3LTljYjMtNGVlYy1iYTI1LWRmM2MzYmVlM2M2YyIsImRhdGEiOnt9LCJyYW5kb20iOiIzNzU0Y2RjMDdmZTlkYzNmMWVmNTJjNTc5NTc4MTZkZiJ9.YAu5Lc4IMsUnTDAfdiiaX0cEFO66kH3-Tc0IBYMtxRf6O824e85uRvD_m2Dip9cDC_IUg2CIxisSaN5iwdyrFQ and https://zenodo.org/records/17274398?token=eyJhbGciOiJIUzUxMiJ9.eyJpZCI6ImRhZWM4ZDMzLWYxNTAtNGM2Ni04ZGQzLWM0YTQ4NmI4YjllOCIsImRhdGEiOnt9LCJyYW5kb20iOiI4OWE2MGMyMjFmYzFhMzBiZjYxNTJkYTMwYjQxZGJmOSJ9.22PVvcpoKy7oAhugaqx5iSgAL2v0DMbGO-obROSh16bxkeHw-eLIbeIfibTVjZ_1klseB8EH2NhfVPCAbyMygA. Further inquiries can be directed to the corresponding author.”

The original article has been updated.

